# DNA-templated synthesis of PtAu bimetallic nanoparticle/graphene nanocomposites and their application in glucose biosensor

**DOI:** 10.1186/1556-276X-9-99

**Published:** 2014-02-27

**Authors:** Jing Leng, Wen-Min Wang, Li-Min Lu, Ling Bai, Xin-Lan Qiu

**Affiliations:** 1 College of Science, Jiangxi Agricultural University, Nanchang 330045, People's Republic of China

**Keywords:** Graphene, PtAu bimetallic nanoparticles, Glucose oxidase, Biosensor, Glucose

## Abstract

In this paper, single-stranded DNA (ss-DNA) is demonstrated to functionalize graphene (GR) and to further guide the growth of PtAu bimetallic nanoparticles (PtAuNPs) on GR with high densities and dispersion. The obtained nanocomposites (PtAuNPs/ss-DNA/GR) were characterized by transmission electron microscopy (TEM), energy-dispersive X-ray spectrometer (EDS), and electrochemical techniques. Then, an enzyme nanoassembly was prepared by self-assembling glucose oxidase (GOD) on PtAuNP/ss-DNA/GR nanocomposites (GOD/PtAuNPs/ss-DNA/GR). The nanocomposites provided a suitable microenvironment for GOD to retain its biological activity. The direct and reversible electron transfer process between the active site of GOD and the modified electrode was realized without any extra electron mediator. Thus, the prepared GOD/PtAuNP/ss-DNA/GR electrode was proposed as a biosensor for the quantification of glucose. The effects of pH, applied potential, and temperature on the performance of the biosensor were discussed in detail and were optimized. Under optimal conditions, the biosensor showed a linearity with glucose concentration in the range of 1.0 to 1,800 μM with a detection limit of 0.3 μM (S/N = 3). The results demonstrate that the developed approach provides a promising strategy to improve the sensitivity and enzyme activity of electrochemical biosensors.

## Background

It is well known that the diabetes mellitus is one of the leading causes of death and disability in the world which can be easily diagnosed and managed by the determination of blood glucose [1]. The great importance of glucose monitoring leads to a considerable amount of fascinating research and innovative detection strategies. Among various glucose detection methods, such as spectrophotometric [2], chemiluminescence [3], and electrochemical methods [4-6], the amperometric electrochemical biosensor based on glucose oxidase (GOD) has played a leading role in the move of simple one-step blood sugar testing. Since the development of the first glucose biosensor, improvement of the response performances of enzyme electrodes has continued to be the main focus of biosensor research [7]. In particular, research for new materials and methods for immobilizing enzyme is still a very important subject to get more active and stable biosensors.

GR, with a two-dimensional (2D) sp^2^-hybridized carbon structure in a single-atom-thick sheet, has rapidly emerged as one of the most attractive materials [8,9]. Due to its unique physical and chemical properties, such as high surface area, excellent conductivity, good chemical stability, and strong mechanical strength, GR provides an ideal base for electronics, electric devices, and biosensors [10-17]. Recently, GR-based hybrids are of scientific and industrial interest due to the synergistic contribution of two or more functional components. With appropriate designs, nanocomposites can exhibit the beneficial properties of each parent constituent, producing a material with improved performance. Up to now, various materials have been incorporated with GR layers, including conducting polymers [18], carbon nanospheres [19], metal nanoparticles (NPs) [20], and ionic liquid [21], to construct electrochemical sensors. Among them, metal NPs have received a great deal of interest on account of their unique electronic, chemical, and optical properties. Because PtNPs and AuNPs could provide a suitable microenvironment for biomolecule immobilization and facilitate electron transfer between the immobilized protein and PtNPs and AuNPs, they have been widely applied in immunosensors and biosensors [22-24]. On the basis of the outstanding physical and chemical properties of PtNPs, AuNPs, and GR composites, it is highly desirable that a hybrid composed of PtAu bimetallic nanoparticles (PtAuNPs) and GR could be used as the sensing platform in electrochemical biosensors.

To date, GR-metal hybrids are primarily prepared by *in situ* growth method. However, it is difficult to grow small and uniformly distributed metal NPs on GR surface. In addition, the resulting GR-metal hybrids are mostly in the form of precipitate and not suitable for applications requiring well-dispersed materials. In order to obtain water-soluble GR-based hybrids, various molecules including polymers and surfactants have been recently utilized to functionalize GR [25,26] as supports for metal NPs, but great challenges still remain in rationally functionalizing GR as a superior support for significantly improved electrochemical performance. Deoxyribonucleic acid (DNA) is a well-known natural biological macromolecule, which has regularly arranged functional groups and well-developed chemistries for different specific modifications [27]. Recently, the combination of DNA with carbon-based nanomaterials such as carbon nanotubes (CNTs) through *π*-stacking for the development of novel biomaterials and devices has attracted great attention in the field of DNA transporters [28] and field-effect transistors [29]. Also, DNA can be used as an inexpensive, well-characterized, controllable, and easily adaptable material to construct defined hybrid nanostructures [30,31]. Therefore, DNA modification is expected to eliminate the aggregation of GR for high dispersion efficiency, and its well-developed chemistries may direct the growth of metal NPs with uniform distribution on GR.

**Figure 1:**
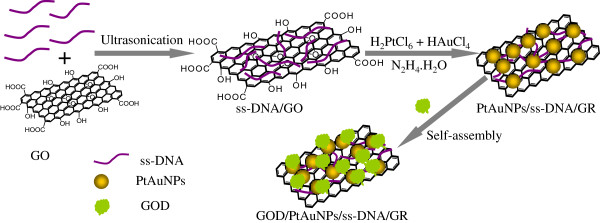
The formation procedures of GOD/PtAuNP/ss-DNA/GR nanocomposites.

In this paper, an amperometric glucose biosensor based on GOD/PtAuNP/ss-DNA/GR nanocomposite was developed. Single-stranded DNA (ss-DNA) was employed to functionalize GR-forming ss-DNA/GR nanocomposite via noncovalent *π*-*π* conjugation between the base pairs of DNA and GR. The ss-DNA bonded to the GR could provide addresses for localizing Au(III) and Pt(IV) along the GR. Then, using a simple chemical reduction method, PtAuNPs were assembled onto ss-DNA/GR with high uniformity and controlled densities. The GOD enzymes were immobilized on the surface of PtAuNP/ss-DNA/GR nanocomposites as shown in Figure 1. The nanocomposites provided a suitable microenvironment for GOD to retain its biological activity. The direct and reversible electron transfer between GOD and the hybrid electrode was observed. The proposed biosensor had good performances in the determination of glucose at a low applied potential with wide linear range, low detection limit, good selectivity, stability, and reproducibility.

## Methods

### Experimental device and reagent

A transmission electron microscopy (TEM) image was taken with a JEM-3010 transmission electron microscope (JEOL Co., Ltd., Tokyo, Japan). The cyclic voltammetric, amperometric, and electrochemical impedance spectroscopy measurements were carried out on a CHI 760B electrochemical workstation (CH Instruments, Inc., Shanghai, China). Electrochemical impedance spectroscopy was performed in a 5 mM K_3_Fe(CN)_6_/K_4_Fe(CN)_6_ (1:1) mixture with 0.1 M KCl at a formal potential of 240 mV using an alternating voltage of 5 mV. The frequency range was from 1 Hz to 100 kHz. A three-electrode cell (10 mL) was used with the modified glassy carbon (GC) electrode as the working electrode, a saturated calomel electrode (SCE) as the reference electrode, and platinum foil electrode as the counter electrode. All potentials were measured versus the SCE, and all experiments were carried out at room temperature.

Native double-stranded DNA (ds-DNA) from calf thymus and GOD were purchased from Sigma Chemical (St. Louis, MO, USA). Graphite powder (99.95%, 325 mesh), hydrogen peroxide solution (30 wt.% aqueous), and hydrazine solution (50 wt.%) were purchased from the Beijing Chemical Reagent factory (Beijing, China) and used as received. All other reagents were of analytical grade, and double-distilled water was used throughout the experiments.

### Preparation of graphite oxide, ss-DNA/GR, and PtAuNP/ss-DNA/GR nanocomposite

Graphite oxide (GO) was prepared from graphite powder according to the method of Hummers [32], and the PtAuNP/ss-DNA/GR nanocomposites were synthesized according to the reported method with a slight modification [33]. Briefly, an aqueous solution of ds-DNA was first heated at 95°C for 2 h to obtain an aqueous solution of ss-DNA. GO (60 mg) was dispersed in water (60 mL) containing 6 mg mL^-1^ ss-DNA by ultrasonic treatment for 30 min. Then, a 0.02 M H_2_PtCl_6_ and 0.02 M HAuCl_4_ solution was added and stirred for 30 min. The mixture was then heated to reflux at 100°C for 4 h to prepare the PtAuNP/ss-DNA/GR nanocomposite. After cooling to room temperature, the resulting materials were then centrifuged and washed three times with distilled water. The as-prepared PtAuNP/ss-DNA/GR nanocomposite was water soluble and could be stored as an aqueous solution at a concentration of 2 mg mL^-1^. Additionally, the preparation of ss-DNA/GR, PtNP/ss-DNA/GR, and AuNP/ss-DNA/GR composites was done in a similar procedure except that there was no addition of H_2_PtCl_6_ or HAuCl_4_.

### Fabrication of GOD/PtAuNP/ss-DNA/GR modified electrode

To prepare the enzyme-modified electrode, a bare GC electrode was polished to be mirror-like with alumina powder (0.05 μm), then washed successively with double-distilled water, anhydrous ethanol, and double-distilled water in an ultrasonic bath, and was dried under N_2_ before use. In order to accomplish electrode coating, 5- μL aliquots of the PtAuNP/ss-DNA/GR solution were dropped and dried on the surface of a GC electrode. The PtAuNP/ss-DNA/GR-modified electrode was then immersed in a GOD working solution (10 mg mL^-1^, 0.1 M PBS) for about 8 h at 4°C to immobilize GOD on the surface of the electrode (Figure 1). Finally, the fabricated glucose biosensor (GOD/PtAuNPs/ss-DNA/GR) was rinsed thoroughly with water to wash away the loosely adsorbed enzyme molecules. The fabricated glucose biosensor was stored at 4°C in a refrigerator when not in use. For comparison, GOD/PtNPs/ss-DNA/GR, GOD/AuNPs/ss-DNA/GR, and GOD/ss-DNA/GR were prepared through similar procedures.

## Results and discussion

### Characterization of ss-DNA/GR and PtAuNP/ss-DNA/GR nanocomposites

**Figure 2:**
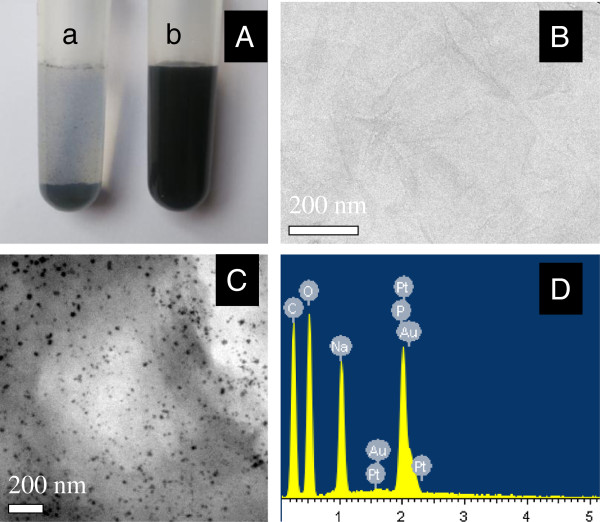
**Photographic and TEM images and EDS spectra. (A)** Photographic images of (a) unmodified GR and (b) ss-DNA/GR in water. TEM images of **(B)** ss-DNA/GR and **(C)** PtAuNP/ss-DNA/GR nanocomposites. **(D)** EDS spectra of PtAuNP/ss-DNA/GR nanocomposites.

GR, chemically derived from graphite oxide, cannot be well-dispersed in aqueous solution due to its hydrophobic nature, so it always forms agglomerates with badly ordered architectures. As shown in Figure 2A(a), GR agglomerates are completely settled down at the bottom of the vial from aqueous solution immediately after removal of the sonication probe, thus leaving the supernatant colorless. By contrast, the ss-DNA/GR suspension even at a concentration as high as 2 mg mL^-1^ appears to be very homogenous and stable (Figure 2A(b)). A TEM image of the as-prepared ss-DNA/GR and PtAuNP/ss-DNA/GR nanocomposites is shown in Figure 2B,C. As can be seen in Figure 2B, the ss-DNA/GR sheets were crumpled and wrinkled on the substrate, which provided an ideal matrix for the distribution of bimetallic NPs. In Figure 2C, the uniform PtAuNPs were well dispersed on the ss-DNA/GR sheets, which might be attributed to the oxygen-containing functionalities on the surface of ss-DNA [34]. In addition, the composition of PtAuNP/ss-DNA/GR nanocomposites was analyzed by energy-dispersive X-ray spectrometer (EDS) (Figure 2D). It shows that the PtAuNP/ss-DNA/GR nanomaterials were composed of C, O, Na, P, Pt, and Au elements.

### Electrochemical impedance spectroscopy characterization of self-assembly process

**Figure 3:**
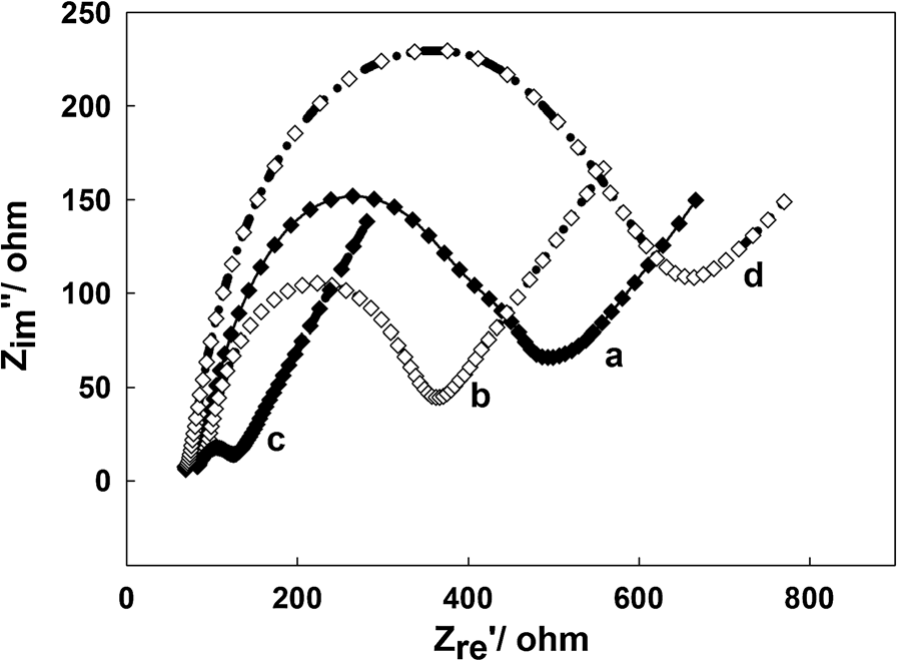
**Impedance spectrum of various electrodes in 5.0 mM K**_**3**_**Fe(CN)**_**6**_**/K**_**4**_**Fe(CN)**_**6 **_**(1:1) containing 0.1 M KCl.** Bare electrode (curve a), ss-DNA/GR modified electrode (curve b), PtAuNP/ss-DNA/GR modified electrode (curve c), and GOD/PtAuNP/ss-DNA/GR modified electrode (curve d).

In electrochemical impedance spectroscopy measurements, the semicircle diameter of impedance equals the electron transfer resistance (Ret), which controls the electron transfer kinetics of the redox probe at the electrode interface and is an important parameter. Figure 3 presents the representative impedance spectrum of the bare electrode (curve a), ss-DNA/GR modified electrode (curve b), PtAuNP/ss-DNA/GR modified electrode (curve c), and GOD/PtAuNP/ss-DNA/GR modified electrode (curve d) in 5.0 mM K_3_Fe(CN)_6_/K_4_Fe(CN)_6_ (1:1) containing 0.1 M KCl. When ss-DNA/GR was modified onto the bare electrode (curve b), the semicircle decreased distinctively compared with the bare GC electrode (curve a), which might be attributed to the excellent conductivity of ss-DNA/GR. The immobilized PtAuNPs on the ss-DNA/GR modified electrode (curve c) made the semicircle decrease again, indicating that PtAuNPs could accelerate the electron transfer between the electrochemical probe [Fe(CN)_6_]^3-/4-^ and the GC electrode. After GOD assembled on the PtAuNP/ss-DNA/GR electrode (curve d), the semicircle dramatically increased, indicating that the presence of the GOD molecules on the electrode surface blocked the electron transfer.

### Electrochemical properties of GOD/PtAuNP/ss-DNA/GR modified electrode

**Figure 4:**
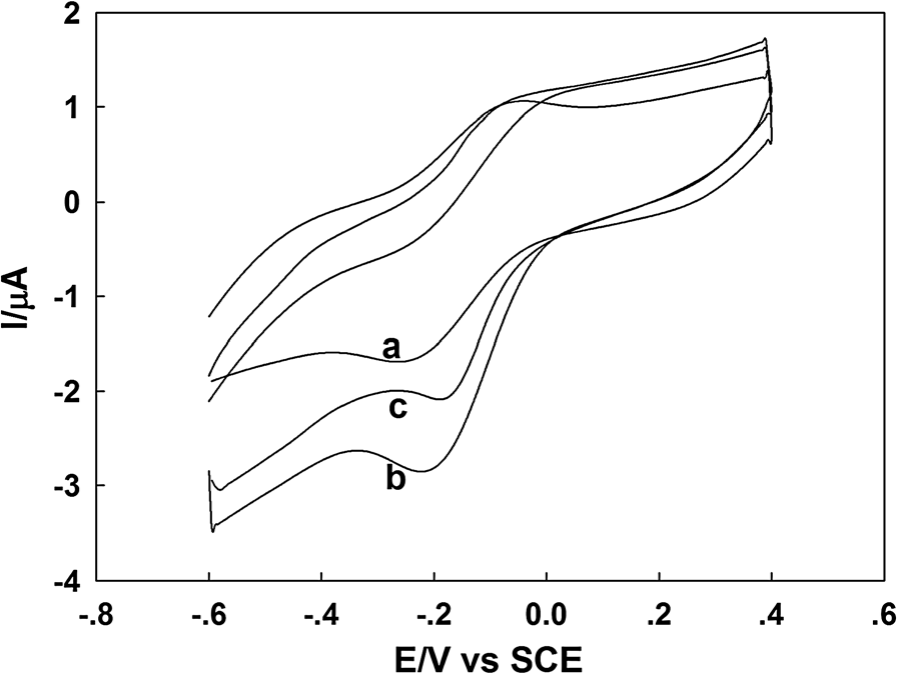
**Cyclic voltammograms of GOD/PtAuNP/ss-DNA/GR modified electrode.** They are in (curve a) N_2_-saturated and O_2_-saturated PBS (pH 7.0) in the (curve b) absence and (curve c) presence of 1.0 mM glucose at 100 mV s^-1^.

Figure 4 shows the cyclic voltammograms (CVs) of GOD/PtAuNP/ss-DNA/GR modified electrode in N_2_-saturated PBS (curve a), O_2_-saturated PBS without 1.0 mM glucose (curve b), and O_2_-saturated PBS containing 1.0 mM glucose (curve c). As shown in Figure 4 (curve a), a pair of symmetrical redox peaks in the N_2_-saturated solution was observed, indicating that GOD undergoes a reversible electrochemical reaction. The shape of redox peaks for the direct electron transfer of GOD dramatically changed in the presence of O_2_ (Figure 4 (curve b)) as the reduction peak current increases, whereas the oxidation peak current decreased. The changes in anodic and cathodic peaks confirmed that GOD in the GOD/PtAuNP/ss-DNA/GR modified electrode catalyzed the reduction of O_2_[35]. The electrocatalytic process of GOD/PtAuNP/ss-DNA/GR modified electrode is expressed as follows [36]:

(1) $$\mathrm{GOD}\;\left(\mathrm{FAD}\right)+2H^{+}+2e^{-}\to \mathrm{GOD}\;\left({\mathrm{FADH}}_{2}\right)\;$$

(2) $$\mathrm{GOD}\;\left({\mathrm{FADH}}_{2}\right)+O_{2}\to \mathrm{GOD}\;\left(\mathrm{FAD}\right)+H_{2}O_{2}$$

where GOD (FAD) and GOD (FADH_2_) represent the oxidized and reduced form of GOD, respectively.

Upon addition of 1.0 mM glucose into the PBS (Figure 4 (curve c)), the reduction peak current decreased. This can be attributed to the decrease in O_2_ content of the solution as it is consumed during the oxidation of glucose by the immobilized GOD. The mechanism for the electrode response process could be expressed as the following reaction [37]:

(3) $$\begin{array}{ll} \mathrm{GOD}\;\left(\mathrm{FAD}\right) & +\mathrm{Glucose}\to \mathrm{GOD}\;\left({\mathrm{FADH}}_{2}\right)\\ & +\mathrm{Gluconolactone} \end{array}$$

According to the reaction above, there is a linear relationship between the amount of glucose increase and the dissolved O_2_ decrease, that is, a model of the glucose amperometric biosensor could be constructed by detecting the decrease of the reduction peak current of dissolved O_2_ to indicate the concentration of glucose.

### Optimization of experimental conditions

**Figure 5:**
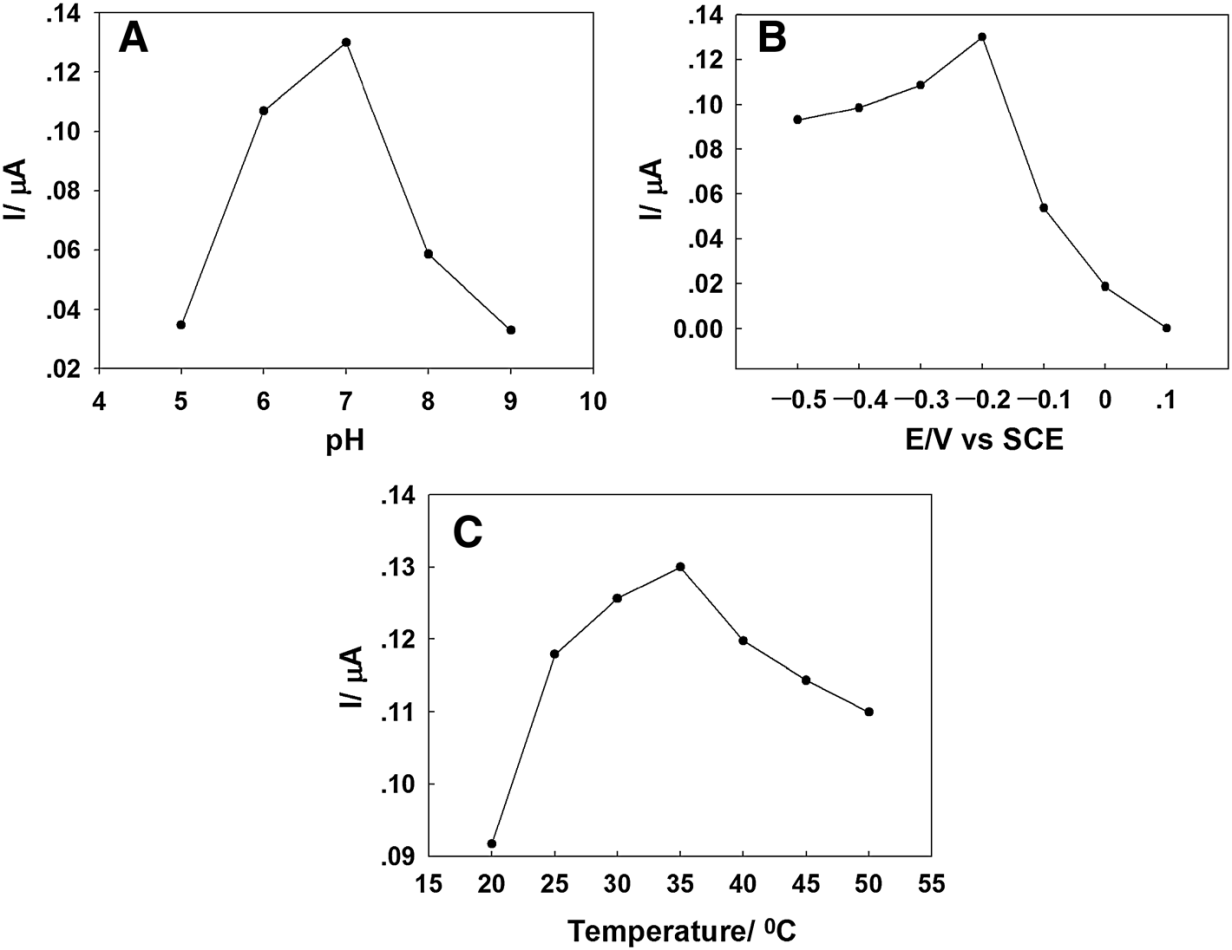
**Effects of (A) pH, (B) applied potential, and (C) temperature.** These are effects on amperometric response of the GOD/PtAuNP/ss-DNA/GR modified electrode to 0.1 mM glucose in 0.1 M PBS (pH 7.0).

The pH value is one of the parameters that affect the response of GOD/PtAuNP/ss-DNA/GR modified electrode to glucose. Figure 5A presents the pH dependence of the amperometric response of 0.1 mM glucose in the pH range of 5.0 to 9.0 at the potential of -0.2 V. It can be seen that the current increased as the pH changed from 5.0 to 7.0 and then decreased above pH 7.0. The maximum response was obtained at pH 7.0, which was consistent with the previously reported GOD-based modified electrode [37,38]. Therefore, a pH 7.0 PBS was used as the electrolyte in subsequent experiments.

The applied potential is an important parameter that affects the sensitivity of the biosensor. Figure 5B displays the dependence of applied potential on the amperometric response of the biosensor to 0.1 mM glucose in PBS (pH 7.0). When the applied potential was changed from 0 to -0.35 V, the maximum response current was observed at -0.2 V. To obtain high sensitivity and to minimize possible interferences, -0.2 V was chosen as the optimum applied potential for further investigations.

The effect of temperature on the amperometric response of glucose was also studied. The biosensor was immersed into the buffer solution at a given temperature for 10 min before amperometric detection, and then the response of the electrode was measured at this temperature. As shown in Figure 5C, the electrochemical response increases with increasing temperature from 25°C to 35°C and then decreases as the temperature further increased. The sharp decrease of the response was due to the denaturation of GOD at high temperatures. Although the response of the biosensor was greatest at 35°C, for practical reasons, it was suggested that room temperature be used to simplify the experimental procedure and prolong the useful lifetime of the biosensor given that most enzymes can be easily denatured at high temperature.

### Amperometric sensing of glucose

**Figure 6:**
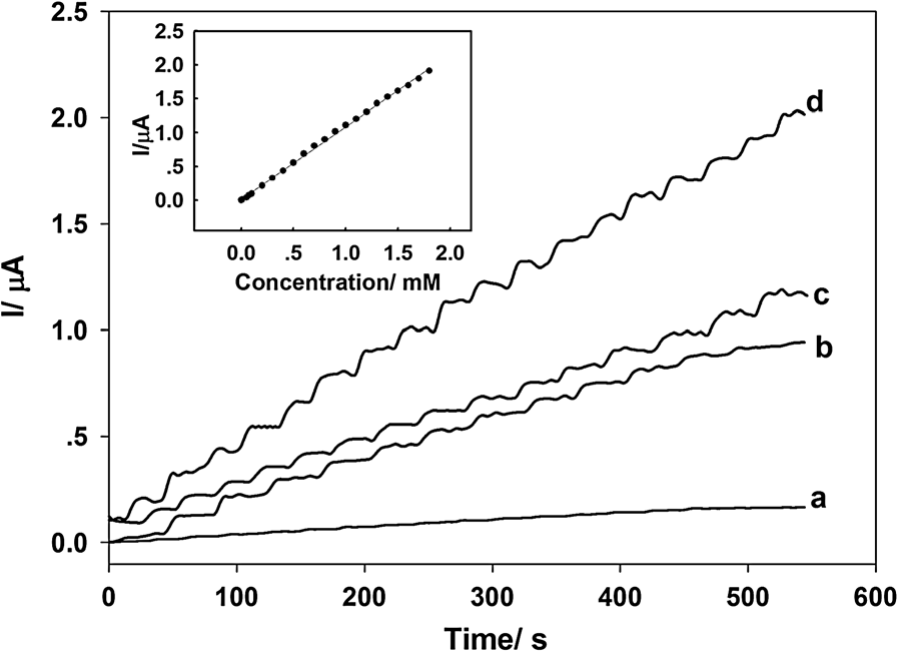
**Amperometric responses of modified electrodes to additions of 0.1 mM glucose in 10-mL PBS at -0.2 V.** GOD/ss-DNA/GR (curve a), GOD/PtNP/ss-DNA/GR (curve b), GOD/AuNP/ss-DNA/GR (curve c), and GOD/PtAuNP/ss-DNA/GR (curve d) modified electrodes. Left inset is the calibration curve of the biosensor.

In this work, PtAuNP/ss-DNA/GR nanocomposites were used to accelerate electron transfer between the electro-active sites embedded in GOD and the modified electrode. To investigate the effect of PtAuNP/ss-DNA/GR on the response current, as in Figure 6, we compared the amperometric responses of GOD/ss-DNA/GR (curve a), GOD/PtNP/ss-DNA/GR (curve b), and GOD/AuNP/ss-DNA/GR (curve c) modified electrodes for the successive addition of 0.1 mM glucose at an applied potential of -0.2 V. It can be seen from Figure 6 that the amperometric responses of GOD/PtAuNP/ss-DNA/GR (curve d) modified electrode were much larger than those of the GOD/ss-DNA/GR (curve a), GOD/PtNP/ss-DNA/GR (curve b), and GOD/AuNP/ss-DNA/GR (curve c) modified electrodes. The reason might be due to the extra active surface area provided by PtAuNP/ss-DNA/GR composites and the synergistic action of PtAuNPs and GR. The GOD/PtAuNP/ss-DNA/GR modified electrode exhibited a linear response in the concentration range of 1.0 to 1,800 μM, with a correlation coefficient of 0.997. It was much wider than that of the ZnO/MWCNT/GOD electrode (6.67 to 1,290 μM) [39], Ag polydopamine@CNT/Nafion/GOD electrode (50 to 1,100 μM) [40], and GR quantum dot/GOD electrode (5 to 1,270 μM) [30]. The detection limit was estimated to be 0.3 μM (based on S/N = 3) for glucose, which was lower than 20 μM for MWCNT-GOD [41], 20 μM for GR-chitosan/GOD [42], and 0.5 μM for polyaniline/CNT/Pt/GOD [43].

### Selectivity, reproducibility, and stability of the biosensor

In the present work, we studied the interference effect of ascorbic acid (1.0 mM), dopamine (1.0 mM), and uric acid (1.0 mM) on the amperometric response of 1 mM glucose, and the response is shown in Table 1. As shown, the biosensor showed excellent selectivity to glucose in the presence of ascorbic acid, dopamine, and uric acid. The good selectivity of this biosensor is largely attributed to the low working potential (-0.2 V).

**Table 1:** Interference studies at the GOD/PtAuNP/ss-DNA/GR modified electrode

**Substrate**	**Response current (μA)**
Glucose (1 mM)	1.11
Ascorbic acid (1 mM)	0.0192
Dopamine (1 mM)	0.0156
Uric acid (1 mM)	Approximately 0

The reproducibility and repeatability of the developed biosensor were determined. In a series of 10 biosensors prepared in the same way, a relative standard deviation (RSD) of 5.1% was obtained toward 0.1 mM glucose, indicating the reliability of the method. A set of 10 different amperometric measurements for 0.1 mM glucose with a single sensor yielded an RSD of 4.6%.

The stability of the glucose biosensor was explored. The proposed biosensor was stored at 4°C in the refrigerator. The response to 0.1 mM glucose was tested each week; after 21 days of storage, the response of the biosensor only had a decrease of 5.5% compared to the initial response, which shows long-term stability. Such a high stability could be attributed to the favorable microenvironment that maintains the GOD activity and prevents the leakage of enzyme.

### Real sample analysis

The practical applications of the designed biosensor were evaluated by the determination of glucose recovery in human blood serum. The recovery was investigated by spiking with different concentrations of glucose to serum sample. The samples were diluted 1,000 times before determination. The analytical results are shown in Table 2. One observed that the results obtained in human blood serum showed good results with average recoveries from 98.5% to 102.5%, which confirmed that the proposed biosensor was applicable for practical glucose detection.

**Table 2:** Amperometric determination of glucose in human blood serum samples

**Sample**	**Added (μM)**	**Found (μM)**	**RSD (%)**^**a**^	**Recovery(%)**
1	50.0	51.2	3.1	102.5
2	100.0	98.5	3.2	98.5
3	150.0	151.9	2.8	101.3

^a^Calculated from three separate experiments.

## Conclusions

In this work, a novel electrochemical GOD biosensor based on PtAuNP/ss-DNA/GR nanocomposites was developed for the determination of glucose. The bionanocomposite film provided a suitable microenvironment, which could effectively present a large loading amount of enzyme and enhanced the direct electron transfer between the enzyme's active sites and the electrode. The modified electrode exhibited excellent analytical performance with wide linear range, low detection limit, and good selectivity for measuring glucose. Therefore, the composite of PtAuNPs/ss-DNA/GR is a good material platform, promising for construction of the third-generation enzyme biosensor, biofuel cells, and bioelectrochemical devices.

## Abbreviations

EDS: energy-dispersive X-ray spectrometer; GOD: glucose oxidase; GR: graphene; PtAuNPs: PtAu bimetallic nanoparticles; ss-DNA: single-stranded DNA; TEM: transmission electron microscopy.

## Competing interests

The authors declare that they have no competing interests.

## Authors' contributions

The manuscript was written through the contributions of all authors, JL, W-MW, L-ML, LB, and X-LQ. All authors read and approved the final manuscript.

## Authors' information

JL is an undergraduate student at Jiangxi Agricultural University. W-MW, L-ML, LB, and X-LQ are teachers at Jiangxi Agricultural University.
